# Caregiver perceptions of a pediatric produce prescription program during the COVID-19 pandemic

**DOI:** 10.3389/fnut.2024.1304519

**Published:** 2024-03-21

**Authors:** Zhongyu Li, Fang Fang Zhang, Sean B. Cash, Kurt Hager, Leo Trevino, Sara C. Folta

**Affiliations:** ^1^Friedman School of Nutrition Science and Policy, Tufts University, Boston, MA, United States; ^2^Amistad Community Health Center, Corpus Christi, TX, United States

**Keywords:** produce prescription programs, qualitative research, pediatric population, caregivers, food is medicine

## Abstract

**Introduction:**

Produce prescription programs are rapidly expanding as a type of Food is Medicine intervention with prospects for mitigating food insecurity and reducing diet-related health disparities. Gaining insight into participant perspectives on program logistics and perceived impacts is crucial to program success and improvements.

**Methods:**

Between May and June 2021, we conducted individual and small group interviews with 23 caregivers with children aged 1–5 years who participated in a produce prescription program from 2020 to 2021 in Texas, U.S. They were provided with a gift card to a major national grocery retailer to purchase fresh produce. The card was reloaded $60 monthly for 8 months with automatic roll-over of unused funds to the next month. Participants also received nutrition education in the form of two videos. A deductive analysis approach was employed, and NVivo qualitative data analysis software was used to perform coding and to assist with subsequent analyses.

**Results:**

All 23 participants were female, with an average age of 37.5 years, and the majority identified as Hispanic/Latino (83%). About 43% of the families had three or more children. Six themes were generated from interviews. Three of these themes were related to program logistics: (1) ease of program use; (2) participant satisfaction with the incentive; and (3) desire for additional store options. The remaining main themes pertained to program impact: (1) the enhanced ability to purchase produce; (2) the usefulness of the nutrition education; and (3) persistent challenges encountered when preparing the produce for picky eaters and young children.

**Conclusion:**

A pediatric produce prescription program was perceived as logistically easy and a helpful source of financial support for accessing fresh produce. Program features such as card-based incentive system and partnership with major grocery retailer were favored by participants. For future program design, it may be beneficial to consider collaborating with multiple grocery outlets and enhancing the intensity and targeting of nutrition education.

## 1 Introduction

Suboptimal diet is a major risk for non-communicable diseases, including obesity, diabetes, cardiovascular disease, and cancer (1–3). In the United States, more than 45% of cardiometabolic-related deaths and $50 billion healthcare costs were attributable to poor diet in 2012 (1, 4). Despite a modest increase in overall dietary quality in the past decades, most Americans do not meet the recommended intake of fruits and vegetables (5–7). About 40% of children aged 2–5 years were estimated to have poor dietary quality according to the 2015–2016 National Health and Nutrition Examination Survey (NHANES) (8).

Moreover, diet-related disparities have worsened across multiple sociodemographic factors, especially by race, education, and economic status (7–9). These populations are also disproportionately affected by food insecurity, defined as inadequate access to sufficient and nutritious food for an active and healthy life. In 2021, U.S. households with children less than 6 years old had a higher rate of food insecurity (12.9%) than the national average (10.2%) (10). Compared with non-Hispanic White households with children, Black, Asian and Hispanic households with children were at increased risk for food insecurity and the gap widened since the onset of the COVID-19 pandemic (10, 11). Food insecurity is associated with compromised dietary quality, poor health outcomes, and negative implications for child development independent of poverty (12, 13). Children growing up in food-insecure environments are more likely to experience co-existing health consequences of inadequate nutrient intake and obesity (13, 14).

Food-insecure households often lack the financial and physical resources to access healthy foods like fresh produce and whole grains (15). Federal nutrition assistance programs such as the Supplemental Nutrition Assistance Program (SNAP), the Special Supplemental Nutrition Program for Women, Infants, and Children (WIC), and school meals programs may alleviate hunger and food insecurity by providing resources to meet food needs. A national survey conducted by the U.S. Department of Agriculture showed that about 56% households experiencing food insecurity participated in one of the major nutrition assistance programs in 2021 (10). Additionally, it has been estimated that school meals (breakfast and lunch) may contribute to more than 40% of daily energy and nutritional needs for children living in low-income households (16). Yet, how these programs affect dietary quality and intake of specific food groups remain inconclusive. In fact, several studies observed no differences in fruit and vegetable intakes comparing program participants with income-eligible non-participants among U.S. children, highlighting potential areas for more targeted interventions (17–19).

Produce prescription programs, a type of Food is Medicine intervention, offer participants monetary incentives such as vouchers or debit cards to purchase fruits and vegetables at farmers market or grocery retailers (20–22). Patients who are food insecure and/or at risk of diet related chronic disease are screened and referred to the programs by their healthcare providers. Emerging literature on this type of program indicates that participation is associated with increased produce consumption, reduced food security, and improved cardiometabolic outcomes (20, 23, 24). These programs may present an opportunity to address diet-related health disparities through integrating food and nutrition strategies into the healthcare system. Healthcare providers engaged in these programs have reported that although there were challenges related to staff time, they perceived an increase in knowledge, confidence, and motivation to screen and address food insecurity. They also appreciated that programs provided a tangible way to assist their patients, especially for under-resourced patients who might not qualify for other assistance programs or in times of emergency (25, 26).

Although interest in research and healthcare policy related to food is medicine interventions is growing rapidly, programs prioritizing children are still limited. Only eight studies have aimed to understand the experience of caregivers in pediatric programs in the past decades (27–30). In more recent qualitative studies of pediatric produce prescription programs operated during the COVID-19 pandemic, themes from the perspectives of caregivers revealed exacerbated economic challenges related to the pandemic, adaptations made in response to program implementation, and perceived favorable program effects on healthy food access and family dietary habits (28–30). These previous studies underscored the diversity of experiences with produce prescription program, which could be affected geographic and temporal factors. Policy reports call for more qualitative research on the experiences of program participants to explore the effect of cultural and socioeconomic factors on program adoption as well as to identify program impacts and areas for improvement that are not otherwise captured in quantitative studies (23).

Most prior programs mainly used vouchers for farmers’ market or fresh produce boxes, offering very limited variations on incentive form and redemption partners (e.g., voucher vs. card-based incentives). In this study, we focused on understanding how program participants perceived a card based pediatric produce prescription program that partnered with a main grocery outlet implemented during COVID-19 pandemic in Texas U.S. where more than 20% children faced food insecurity in 2021 (31), how participating in the program affected food access and dietary behaviors, and how programs might be improved to better serve their needs in the future.

## 2 Methods

### 2.1 Study design and sample

In May–June 2021, we conducted three individual and six group interviews (each ranging from two to seven participants) with a total of *n* = 23 caregivers who participated in a produce prescription program from 2020 to 2021 that was run through a community health center serving a low-income community in Texas, U.S. Eligible caregivers had at least one child aged 1–5 years. All 23 participants were female; their mean age was 37.5 years and 83% identified as Hispanic/Latino (see Table 1).

**Table 1:** Texas 2020-2021 produce prescription program participant characteristics (*n* = 23).

Age in years, mean (SD)^1^	37.5 (10.1)
**Gender, *n* (%)**	
Female	23 (100)
**Hispanic, *n* (%)**	
Yes	19 (82.6)
No	4 (17.4)
**Race, *n* (%)**	
White	21 (91.3)
Black/African American	1 (4.3)
Did not say	1 (4.3)
**Children in household, *n* (%)** ^**1**^	
One	4 (18.2)
Two	8 (36.4)
Three	3 (13.6)
Four or more	7 (31.2)
**Marital status, *n* (%)**	
Married	10 (43.5)
Divorced	1 (4.3)
Widowed	1 (4.3)
Separated	3 (13.0)
Never married	6 (26.1)
Member of unmarried couple	2 (8.7)

^1^Missing value, *n* = 22.

Caregivers were provided with a gift card to a major national grocery retailer, restricted to fresh produce purchases, that was reloaded with $60 monthly for 8 months. The primary nutritional educational support materials were the two nutritional education videos created for the program and the participants by the wellness coordinator and coach at the participating clinic. The videos were in English and provided via a private link. The first video was released in the first 3 months of the program, and the second video was released in the last 3 months of the program. The topics were “Do not Tell Your Kids to Eat Fruits and Vegetables!” for the first video (approximately 30 min) and “How to Make Eating Healthy Too Easy” for the second video (approximately 60 min).

#### 2.1.1 Interview procedures

Semi-structured interview guides were developed by the Tufts research team’s qualitative research expert (SCF) with input from research team members; the non-profit organization that implemented the produce prescription program evaluated in this study; and the program coordinator at the partnering clinic. The interview guide consisted of two key topic areas: “program logistics” concerning participants’ perceptions on logistics and setup of the program; and “experiences with the program” concerning participants’ experiences with using the program and program impact. A total of six main questions, each with multiple sub-questions, were included in the interview guide (Table 2).

**Table 2:** Program participant interview guide.

Topic 1: Program logistics
For the first set of questions, I’m going to ask about how the program was set up. Talk about how easy or hard it was to understand how to use the program. What, if anything, helped you to better understand the program? [probes: clinic staff, materials] As you know, the program used a reloadable Walmart gift card. I’d like to ask about using the gift card itself. What worked for you? [probes: delivery system, ease of use] What did not work for you? Describe any problems you had using the card at Walmart. What problems, if any, did you have with the store employees when you were using the card? [probes: they did not know what it was, they treated you poorly] What other stores or places to get food would you have wanted to be part of the program? Tell me the reasons. Tell me your thoughts about the amount of the gift card. What amount would have been more helpful?
**Topic 2: Experiences with the program**
For the next set of questions, I’d like to find out more about your experiences with the program. Think back to when you were deciding whether to do this program. What were you hoping to get out of it? Talk about whether the program helped you in the ways you thought it would. How could the program change to best meet your needs? What, if anything, surprised you about the program? Now, I’d like to hear more about your experiences with the fruits and vegetables that you got as part of the program. What did you usually get with the card? How did you usually use them? Who in your household ate the fruits and vegetables? What did your children think about the fruits and vegetables? How easy was it to get fruits and vegetables that you and your children are most familiar with? Tell me about any new fruits and vegetables that you tried because of the program. What, if anything, surprised you about what you tried? What did your child(ren) think about the new fruits and vegetables? In what ways, if any, did your children change what they thought about any fruits or vegetables? Tell me about the best moments you had when using the fruits and vegetables from the program. [probes: what made you proud? What was the most fun? What was the best meal you made?] Describe the biggest challenge you had with the fruits and vegetables that you got as part of the program. [probes: children would not eat the food; did not know how to prepare the food; food went to waste] What, if anything, could be added to the program to help you make better use of the fruits and vegetables? Now I’m going to ask about some other parts of the program besides the card. Tell me your thoughts about the educational videos. What were you hoping to learn from them? What, if anything, did you learn that was new? What other topics would have been helpful? What did you think about using a video to receive this information? Some people were asked to set goals at the beginning of the program. What can you tell me about any goals you set? How helpful was it to set goals? Overall, what did you like best about this program? What about this program was the most challenging? Talk about whether you would do this program again. If you were going to do the program again, what would you do differently? If you were trying to get a friend or family member to join a program like this, what would you tell them? In what ways, if any, did doing this program affect your experience in the clinic? How did the pandemic affect how often you come to clinic? What could the clinic staff have done to make the program better for you? How did your experience with this program compare to any other food programs that you have done? [probes: WIC, food pantries, summer meals for kids]

Interviews (individual and small group) were conducted online using the Zoom videoconferencing application either by the qualitative expert or by study team members whom she had trained. Verbal consent was obtained at the beginning of each interview session. Interviews lasted 21–61 min. All interview sessions were recorded and transcribed verbatim. Study participants received a $50 gift card for remuneration. The protocol was reviewed and deemed exempt by the Tufts University Health Sciences Institutional Review Board.

#### 2.1.2 Analysis

The interviews were recorded, transcribed, and coded using NVivo qualitative data analysis software. We used a directed qualitative content analysis approach, which is fundamentally deductive (32). We drafted initial codebooks based on the interview guides. We then conducted reviews of the transcripts and added codes for topics that arose in the data. Once the codebooks were established, the qualitative expert and the study administrator (ZL) independently coded one transcript. Inter-rater reliability testing identified minor differences in interpretation; coding decisions were reviewed line-by-line and resolved. The codebooks were revised accordingly, mainly by clarifying code definitions, and all transcripts were subsequently coded by the study administrator. Final themes were reviewed and refined by the study team and presented at monthly investigator meetings.

## 3 Results

Six themes were identified, three in each of the two key topic areas of the interview guide—Program Logistics and Program Impact.

### 3.1 Program logistics

Overall, program participants had positive perceptions about the logistics of the produce prescription program. There were three major themes related to participants’ experiences: (1) ease of use; (2) satisfaction with the incentive; and (3) desire for additional store options.

#### 3.1.1 Ease of use

In this program, participants received a gift card to a major national grocery retailer that automatically reloaded $60 each month. Any remaining balance rolled over into the next month. The gift card could be used for any fresh fruits or vegetables. The major theme was that the program and the card was easy to understand and use. Many participants used it at self-checkout, just like any gift or debit card.

*“… it was easy. You just go in, select what you want, go and check out. Rather you have other merchandise or whatever, it would already separate it, it would pay for what it knew it was supposed to take off, so it was just super easy. I was surprised how easy it would be” [white female, 41-year-old, with one child].*

*“I believe it was easy. You would get a text message saying when it was loaded. You were able to call to figure out how much you still had left. Yeah it was simple. It was not hard at all” [white female, Hispanic, 25-year-old, with four children].*

*“I didn’t have any problems with the setup. I heard about it. I contacted my contact at the at [clinic] he got right back with me. And any emails I got on what was to be expected, and what I was supposed to do was very easy to understand and follow” [white female, 44-year-old, with two children].*

Participants perceived store employees as friendly and helpful with the produce prescription. Only a few participants had issues with the program, and any questions were addressed quickly and satisfactorily by the program coordinator at the partnering clinic. A few had issues using the card the first time, but everything went smoothly after they figured out how to use it and what was covered by the card.

*“In the beginning, it was trying to figure it out and everything, but yes. The fruit and vegetables, I didn't know even to get the ones that were bagged or just plain without having them bagged, but it was easy, except in the beginning. When I used it twice and then after that, it was fine” [white female, Hispanic, 60-year-old, with one child].*

A subtheme was that the program was logistically easier than the Special Supplemental Nutrition Program for Women, Infant, and Children (WIC). Participants described several advantages to the program, such as being able to use the card at the self-check-out (which they could not do with WIC benefits), not needing to look for specific items like WIC tagged items when purchasing fruits and vegetables and being able to roll the incentive over to the next month. And a few also mentioned quicker responses to their questions and feeling less judged or embarrassed when using produce prescription card than WIC.

*“Using that card self-checkout was a lot easier because whenever we have to use WIC, we cannot go to self-checkout, you have to go to the cashier. Sometimes, you’re just going for fruits and vegetables, you can just go straight to your self-checkout and it's a lot easier to use that” [white female, Hispanic, 35-year-old, with six children].*

#### 3.1.2 Satisfaction with the incentive

A second major theme was that participants were satisfied with the incentive amount. They described the amount as appropriate and useful, regardless of household size.

*“It was a good amount. For me, it's just me and my son, but he is a big fruit lover. He loves all kinds of fruits. So I would use it every month and he really enjoyed it also” [white female, Hispanic, 28-year-old, with one child].*

*“I think the amount was a really good amount. We're a family of five, now six, but that was, I think more than enough. Especially with the fruits and vegetables, sometimes they can be costly, but I think it was a good amount for us” [female, Hispanic 29-year-old, with four children].*

*“Yes, and I agree also. The vegetables, not so much the vegetables, but I think some of the fruits can be a little bit more pricey during certain seasons, so it was very helpful with that amount” [white female, Hispanic, 41-year-old, with five children].*

Many participants stated that the incentive allowed them to purchase produce with less financial stress, especially when produce prices were high or when income had been reduced due to the effects of the COVID-19 pandemic. Moreover, many participants appreciated that the program provided extra funds specifically focused on fruits and vegetables. Participants also appreciated the flexibility of the incentive, allowing them to make their own choices of fruits and vegetables based on their preferences and needs.

*“I personally love that we had choices, and we could make our own choice. We didn't just … here's a bag of food or here's your meals for the week. We could pick and choose what we wanted, what we liked. We didn't have to go and turn around and pass on the blessing to somebody else because we just can't utilize it because nobody likes it, or there's allergy, or whatever. I really enjoyed being able to pick and choose what we got” [white female, Hispanic, 35-year-old, with two children].*

The only major issue with the incentive that participants described was that it could not be used on frozen fruits and vegetables.

*“The only downfall that I personally wished would be maybe frozen fruits and vegetables included, that would have been ideal because I noticed that sometimes I was like I want to go get them, but not, I don't think they're going to be eaten as much this weekend or whatever, because of our schedule and knowing that it was more of a chance of the produce going bad, not to mention me personally, [participating grocery store] doesn't have the best produce” [white-female, Hispanic, 39-year-old, with six children].*

#### 3.1.3 Desire for additional store options

A third major theme was that more stores should be included in the program. Participants cited several reasons for wanting more options, including convenience and a better selection and quality of produce.

*“I definitely think it would have been an improvement had it been contracted with somebody else like [statewide grocery store] or in addition to. That would have been really beneficial, in my opinion. Just because the quality of the produce really and the selections” [white female, Hispanic, 35-year-old, with two children].*

*“I honestly prefer [health food store], like their fruits. I wish that would have been an option” [white female, 32-year-old, with two children].*

*“Personally, I get all my groceries from [statewide grocery store], so I would have to make a separate trip in order to go to [participating grocery store], which isn't close to the [statewide grocery store] that I go to, but have to make an additional trip in order to get the fruits and veggies because I couldn't justify getting them at [statewide grocery store] and paying for them when I could get more and use the card” [white female, Hispanic, 36-year-old, with three children].*

Only a few other logistical factors with the program were mentioned. A few participants suggested having a program app where the incentive balance and nutrition education could be easily accessed. Related to COVID-19 concerns, some participants stated that being able to purchase produce online and having access to mental health videos or support would have been helpful.

### 3.2 Program impact

Most participants said that all members in the household ate and benefited from the fruits and vegetables purchased using the program. There were three main themes related to program impact. The first was the ability to purchase a greater quantity and variety of fruits and vegetables; a second was the usefulness of the nutrition education; and a third was challenges related to preparing the produce so that it was acceptable to children.

#### 3.2.1 Purchasing a greater quantity and variety of fruits and vegetables

The overarching theme was that participants were able to purchase more produce because of the program. The program enabled participants to purchase more fruits and vegetables that they or the family liked and would normally buy.

*“My little one loves bananas and eats one every day. Whereas before, I would purchase them a little bit more sparingly, I purchased it more because we had the extra amount to use” [white female, Hispanic, 36-year-old, three children].*

*“My kids enjoyed it as well. My oldest is six and my youngest is one. In between, there's a two-year-old and a five-year-old. But they would love to pick their own fruit. They miss being able to get so much at one time. They would pack their lunch bags with fruits and vegetables instead of candy and chips. They really liked it, especially my daughter with the carrots and her ranch. I don't know where it went!” [white female, Hispanic, 25-year-old, with four children].*

*“Every time I would tell my son I'm going to go to [participating grocery store], he would always already know that it was going to be like we're going to go buy fruits and vegetables. He really enjoyed being able to get the fruits he wanted, especially he loves grapes. [laughs] He already knew he could get, instead of just getting one bag, he could get two. He loved it” [white female, Hispanic, 28-year-old, with one child].*

The program allowed participants to purchase a wider variety of produce, and to be adventurous in trying new types. It also allowed participants to buy more produce that they knew their families enjoyed, but that were usually too expensive. For instance, people used the card to purchase more expensive items like cherries and avocados that they would typically buy in small quantities or not at all. A few participants also mentioned buying more higher-quality produce.

*“Yes, we’re exploring different things. Like the coconut, they hadn’t been exposed to the coconut. Different bananas that they have, different carrots that they have; I was definitely able to think outside the box and try something different” [white female, Hispanic, 44-year-old, with seven children].*

*“We were able to get better greens. Then also, I enjoyed that if my son wanted the plums or something, I wasn't just looking at the price and be like, ‘No, we're only going to get the apples that are on sale this week.’ I could get the ones that were 50 cents more a pound and not feel guilty that I was taking away some other food item from our grocery budget that I wasn't going to be able to get that week to make our meals. They were so excited that they could go pick their fruits and vegetables out without worrying about mom being like, ‘No, let's get the cheaper ones’ [laughs] So that was nice” [white female, 32-year-old, with two children].*

#### 3.2.2 Nutrition education

In this program, nutrition education consisted mainly of two educational videos. Participants described learning new ways to add more fruits and vegetables to their children’s diets. Some additionally mentioned new cooking tips and the benefits of consuming fruits and vegetables. Several participants commented that they were already familiar with the video content but felt that it served as a useful reminder.

*“I thought the videos were a good resource for reminders for me. Life gets so busy, and then I would see the messages, so I'd be like you know what, I'm going to sit down for a second and watch this. And then I'd be like okay, now I need to get moving, get back on track, quit eating chips woman, and go and get something else healthier, you know. Just like a -- when the video is there I’d be like oh yeah, I'm trying to do better, I'm not trying to snack on all this garbage all the time” [white female, 41-year-old, with one child].*

*“I enjoyed them. I thought he did a good job presenting the material. They weren't long, it wasn't a drawn-out, long, boring video. It's things that are important, like dealing with picky eaters, and hiding the vegetables. I know there was a portion on one of the videos about exactly what I'm trying to do right now. They were very pertinent to what I was doing at home” [white female, Hispanic, 35-year-old, with two children].*

While the overall theme was on the helpfulness of the nutrition education, several participants had contrasting perspectives. One participant with very young children said that the tips given in the videos were not helpful in getting her kids to eat more fruits and vegetables and try new things. A few participants did not watch or watched only one video. They were either too busy or did not feel motivated to do so.

#### 3.2.3 Challenges

Despite the increased access to fruits and vegetables, a third theme was that participants still sometimes struggled with ways to prepare fruits and vegetables so that their family members would like and eat them. Several participants expressed a desire for new recipes and cooking tips, as well as how to properly feed very young children.

*“I think, well, for me, trying to cook vegetables for my granddaughter is real hard because she's a picky eater. She'll eat cheese, she'll eat crackers, she'll eat almost every fruit there is. Vegetables are just really hard…We can't hide the green. I was thinking just now, maybe I should try to hide cauliflower in there because that's white. I think that would be the most challenging thing that I found, was trying to hide something. It was easier just to give her fruit and cheeses because we knew she would eat that. At two and three years old, the fight is just not there for me anymore” [white female, Hispanic, 59-year-old, with one child].*

*“The biggest challenge for me, the vegetable, it was asparagus, the spinach, and the broccoli and all like that it's pretty cool because I could put it in the smoothies, but the asparagus, I cannot. I try to grill them, I try to put them in salads and grill them or bake them, that's a challenge still right now, ongoing. But she will eat some. One of these days she's going to like them. I'm going to prepare, and they'll like them” [white female, 60-year-old, Hispanic, with one child].*

## 4 Discussion

This qualitative study explored caregivers’ perspectives on a pediatric program implemented during the COVID-19 pandemic among under-resourced families in Texas. In general, parents found the program to be logistically easy, with incentives perceived as adequate, regardless of household sizes. Moreover, program participants shared their experiences of reduced financial obstacles to acquiring fresh produce and an enhanced capacity to purchase a wider variety of fruits and vegetables. Participants expressed that maximizing choice, including the types of produce offered (i.e., frozen in addition to fresh) and ability to access more retail grocery stores was important. Many participants described the experience of the program as easier than WIC and less stigmatizing. Challenges and considerations for future program development included the need for increased grocery store involvement and the provision of recipes.

This study suggests that implementation of produce prescription programs increases purchasing power for fresh produce. Financial constraints have been recognized as a main barrier to access fresh produce among food insecure households, contributing to heightened exposure to poor-quality food, chronic psychological stress, and unhealthy eating behaviors (33). Our findings suggest that produce prescription programs may play a crucial role in alleviating financial stress associated with accessing healthy fresh produce. This perception is shared by other produce prescription participants across several qualitative studies reporting enhanced financial accessibility to healthy produce during the pandemic (28, 29, 34). In future studies of produce prescription programs, quantitative research may confirm the impact of these programs on both perceived financial stress, household food purchases, dietary intake, and health outcomes.

Moreover, our findings align with those of other produce prescription programs, some of which offered lower monthly incentives, yet reported a reduction in the financial barriers to meeting the recommended intake of fruits and vegetables (35, 36). Notably, all participants in our study perceived $60 per month as appropriate and helpful to obtain fresh produce for their families, although family sizes varied from 2 to 9. An effective and sufficient incentive dosage, however, may vary largely by geographic locations and socioeconomic status of participants. More research is warranted in identifying proper incentive amounts to meet the needs of families living with varying conditions and challenges. Additionally, standardizing produce prescription benefits across states through federal legislation or uniform Medicaid guidance could establish minimum standards for pediatric programs, creating consistency and predictability for patients and providers nationwide.

Importantly, participation in this produce prescription program enabled families to explore a greater variety of fruits and vegetables. Studies have shown that consuming a diverse array of fruits and vegetables is associated with better weight management, reduced inflammation, and lower risks of chronic diseases, independently from total intake (37). Both adult and pediatric programs collaborate with key stakeholders like healthcare providers, community organizations, and farmers markets/grocery stores to increase fruit and vegetable consumption and improve health outcomes. However, pediatric programs uniquely target children and families, aiming to cultivate healthy eating habits from a young age and foster family involvement. This early intervention is crucial because exposure to a diversified diet rich in fruits and vegetables fosters positive childhood food experiences, which ultimately contributes to the establishment of long-term healthy dietary behaviors (38, 39).

This is supported by existing research and our own findings. A qualitative study on a fruit and vegetable prescription program among low-income pediatric patients reported children engaging in more frequent tasting and increased acceptance of new fruits and vegetables (40). Moreover, observations by Zimmer et al. indicated that children were more likely to choose fresh fruits over processed snacks after enrolling in a Fresh Food Prescription delivery program (34). In our study, we also observed that parents and children became more adventurous in experimenting with new fresh produce. Produce prescriptions have the potential to facilitate the introduction of healthy foods and eating behaviors in early life and improve long term health outcomes. As such, they may emerge as a valuable strategy within healthcare system to address food and health disparities among children.

Beyond accessibility to fresh produce, nutritional knowledge and food preparation skills can affect the actual utilization and consumption of purchased fruits and vegetables. This becomes particularly crucial for parents with very young children and picky eaters. In both our study and the other qualitative studies of pediatric produce prescription programs, caregivers encountered challenges in preparing fruits and vegetables to get their young family members to try them (34, 40). Therefore, engaging and educating parents or caregivers is fundamental for program success. Incorporating nutrition education as a programmatic component, including food preparation and recipe ideas, may promote the usability of fruits and vegetables that families receive through produce prescriptions (41–43). In this Texas pediatric program, nutrition education was delivered through two 60-min videos due to COVID-19 restrictions, without in-person sessions. Many participants appreciated the usefulness of new perspectives and practical advice on consuming fruits and vegetables, in the convenient format of video that accommodated their busy schedule during the pandemic. However, a subset of participants indicated a desire for additional motivation and content tailored more closely to their specific needs.

A field scan released in 2021 revealed that 70% of produce prescription programs incorporated an educational component that was perceived as helpful in encouraging prescription redemption and produce consumption among participants (22). The report further acknowledged that traditional nutrition education may be insensitive to the existing situation (e.g., nutritional knowledge, culinary skill, and needs) of people whom it serves. Tailoring nutritional education, including cooking tips and recipes, to specific populations such as young children and elders while considering the feasibility and availability of their caregivers as well as effective formats of educational materials, may be needed for produce prescription programs targeting diverse populations to optimize their impact and better address the unique challenges faced by different communities.

Several features of the Texas pediatric program worked well for participants. Participants described advantages to gift cards as a mode of providing the incentive. Program cards helped streamline the check-out process by eliminating the need to separate fruits and vegetables. Participants could conveniently use the card at self-check-out, a feature that many enjoyed and preferred, whereas incentives distributed as vouchers may extend the checkout time to sort exact voucher amounts and eligible items (28, 44). A qualitative study among WIC recipients revealed that major barriers to shopping with WIC included finding eligible items, difficulty at check-out with WIC vouchers, and perceived stigma against WIC participants primarily by cashiers at checkout (45). These barriers may subsequently discourage the utilization of program incentives (44). The program cards, however, were not identified as being related to a specific program, such as SNAP or WIC, which may reduce perceived embarrassment and stigmatization for participants, therefore promoting incentive use and program satisfaction. This highlights the importance of investment in point-of-sale technology in future programming: seamless integration of produce prescription benefits into checkout systems could further streamline enrollment, potentially increase program utilization, and reduce administrative burdens as well as facilitate more robust data collection for program evaluation.

Transportation was not perceived as a consistent or major issue in this study, suggesting that partnering with grocery outlets may make using the incentive more convenient and feasible. Future programs could consider partnering with multiple grocery retailers to increase produce offerings so that participants would be able to choose where to redeem the incentive, which was important to many participants in our study and in others (35, 44, 46). Moreover, this program only covered fresh produce yet expanding eligible items to include minimally processed (e.g., no added sugar) frozen produce, that are as nutritious for meeting the needs of healthy diet, may increase the storability, use of purchase items, less frequent trips to stores (47). For program participants who face challenges in consuming fresh produce quickly enough or desire to minimize their trips to grocery stores due to personal needs or health concerns, inclusion of frozen produce is a good alternative and may enable them to benefit more from the program. These approaches may not only help with any transportation issues but also supports program participants’ valued autonomy and preferences, fostering a positive program experience and potentially higher enrollment and usage.

Providing targeted funding for pilot programs to test and evaluate innovative produce prescription program models in diverse communities including rural areas is critical. These pilots can demonstrate model effectiveness and inform future expansion efforts to promote program accessibility across a wider range of locations and populations. Beyond programmatic components, clinic adoption and sustainability are pivotal factors for program success. Developing workflows for program implementation that align with existing systems, designating specific staff responsibilities related to the program, and scheduling post-enrollment follow-ups with patients can potentially improve operational efficiency and foster clinician engagement.

This study has several limitations. We sampled from one produce prescription program implemented during the COVID-19 pandemic in 2021, which restricts our findings to the unique circumstances and factors specific to this program. It is important to recognize that other programs may operate differently, at different locations and times, and insights from these programs could vary significantly. Moreover, the recruitment process relied on active responses from program participants. Those who responded may have been more actively engaged in the program or otherwise different from those who did not respond. This potential response bias may influence the perspectives captured in our study, potentially reflecting the viewpoints of a specific subgroup of participants. Nonetheless, demographic characteristics such as age, gender (all eligible program participants were female), race/ethnicity, marital status, or number of children in the household did not differ significantly between the 23 study subjects and the 33 who screened eligible for the study but did not participate. This offers some assurance that the data we have were from a group of program participants who were reasonably similar to the entire sample. Still, our findings might not fully represent the experiences and opinions of other groups who may be more marginalized or financially unstable.

## 5 Conclusion

Findings from this study highlight the perceived usefulness of a pediatric produce program prescription program in alleviating financial stress to access fresh produce and promoting fruits and vegetable intake among food-insecure families living in Texas during COVID-19 pandemic in 2021. Program features such as card-based incentive distribution, seamless check-out process and redemption at main grocery retailers were favored by participants. As interests in produce prescription programs continue to grow, future programs should consider partnering with diverse grocery retailers, including frozen produce, and integrating more tailored and targeted nutrition education and culinary support to better accommodate participants’ needs, preferences, and circumstances, ultimately improving the program’s effectiveness and impact.

## Data availability statement

The datasets presented in this article are not readily available because original qualitative datasets will not be shared. Requests to access the datasets should be directed to zhongyu.li@emory.edu.

## Ethics statement

The studies involving humans were approved by Tufts University Health Sciences Institutional Review Board. The studies were conducted in accordance with the local legislation and institutional requirements. The participants provided their oral informed consent to participate in this study.

## Author contributions

ZL: Data curation, Formal analysis, Project administration, Software, Writing – original draft, Writing – review & editing. FZ: Conceptualization, Funding acquisition, Investigation, Writing – review & editing. SC: Conceptualization, Funding acquisition, Writing – review & editing. KH: Investigation, Writing – review & editing. LT: Conceptualization, Investigation, Methodology, Resources, Writing – review & editing. SF: Conceptualization, Data curation, Formal analysis, Funding acquisition, Investigation, Methodology, Software, Supervision, Validation, Writing – review & editing.
